# Supplementary material for: Knowledge of cardiovascular disease risk factors among caregivers of cardiology patients attending Jordan University Hospital
Source: PeerJ. 2024 Jan 31;12:e16830. doi: 10.7717/peerj.16830 (PMC10838082; doi:10.7717/peerj.16830)
Supplement: Supplemental Information 2 [file peerj-12-16830-s002.pdf]

Knowledge of cardiovascular disease risk factors among caretakers of cardiology patients attending Jordan University Hospital

We, a group of students at the University of Jordan - Faculty of Medicine, are conducting this cross-sectional study, which aims to assess the knowledge and understanding of risk factors for cardiovascular disease (CVD) and coronary heart disease (CHD) symptoms among outpatient caretakers attending cardiac clinics at Jordan University Hospital in Jordan.

It only takes 5 minutes to complete this survey

All information provided will be kept confidential and will be used for scientific research purposes only.

* Indicates required question

1. Do you agree to participate in this study *

Mark only one oval.

- Yes
- No

Sociodemographic factors

2. Age Group *

3. Sex

Mark only one oval.

- Male
- Female

4. Education *

Mark only one oval.

- No Formal Education
- Primary
- Secondary
- Bachelor's degree or higher

5. Marital status *

Mark only one oval.

- Single
- Married
- Divorced
- Widowed

6. Occupation *

Mark only one oval.

- Jobless
- Student
- Self-employed
- Employed
- Retired

7. Income *

Mark only one oval.

- Less than 500
- 500-1000
- 1000-1500
- 1500-2000
- more than 2000

8. Residence *

Mark only one oval.

- Urban
- Rural

9. Relationship to Patient *

Mark only one oval.

- Spouse
- Child
- Sibling
- Parent
- Friend

10. Health Insured *

Mark only one oval.

- Yes
- No

11. Perceived health status *

Mark only one oval.

- Good
- Average
- Bad

12. When was the last time you had general health check-ups *

Mark only one oval.

- Never
- Within a year
- Over a year

13. Personal Disease History *

Check all that apply.

- [ ] CVD
- [ ] Stroke
- [ ] Hypertension
- [ ] Diabetes
- [ ] Chronic kidney disease
- [ ] Cancer
- [ ] Chronic pulmonary disease
- [ ] Chronic back pain
- [ ] None

14. Is there a family history of coronary heart disease *

Mark only one oval.

- ( ) Yes
- ( ) No
- ( ) Don't know

15. Is there a family history of deaths of females under 65 years of age or males under 55 years of age due to heart disease? *

Mark only one oval.

- ( ) Yes
- ( ) No
- ( ) Don't know

16. Smoking status *

Mark only one oval.

- [ ] Current (at least 1 cig in the last 6 months)
- [ ] Past (Last cigarette more than 6 months)
- [ ] Never

17. How many days do you exercise per week *

Mark only one oval.

- [ ] I don't exercise
- [ ] Other: __________

18. How many minutes do you exercise per day *

Mark only one oval.

- [ ] I don't exercise
- [ ] Other: __________

19. Weight *

____

20. Length *

____

21. How worried are you about the possibility of having a heart attack? *

Mark only one oval.

- Not worried at all
- Worried
- Extremely worried

Awareness of CVD

22. Answer the following questions based on your knowledge of Coronary heart disease (CHD) *

Mark only one oval per row.

	Yes	No
**Cardiovascular diseases are curable upon completion of described dose.**	⬭	⬭
**CVDs are the leading cause of deaths globally**	⬭	⬭
**Smoking does not increase a risk of CVD**	⬭	⬭
**Diabetes increases ones risk of CVD**	⬭	⬭
**High blood pressure is a risk factor of CVD**	⬭	⬭
**Excessive alcohol drinking is dangerous to cardiovascular health**	⬭	⬭
**High cholesterol in blood prevents one from CVD**	⬭	⬭
**Consuming a lot of vegetables** and fruits	⬭	⬭

and fruits increases the risk of CVD

Consumption of too much salt is a risk to CVD

Having excess body weight increases ones risk of CVD

Regular physical activity will lower a person's chance of getting Cardiovascular disease

Old age is a risk factor for CVD

Men are at higher risk of CVDs compared to women

CVD are not preventable

Stopping smoking will lower chance of heart attack

HDL refers to "good" cholesterol, and LDL refers to "bad" cholestrol

to "bad" cholestrol		
Blood pressure of 140/90 is considered high		
Blood pressure of 140/90 is considered high.		
Nonsmokers can die from second-hand smoke		
Nonsmokers can die from second-hand smoke		

23. Do you think the following complaints are symptoms of a heart attack? *

Mark only one oval per row.

	Yes	No
**Pain or discomfort in the jaw, neck, or back**	⬭	⬭
**Feeling weak, lightheaded, or faint**	⬭	⬭
**Chest pain or discomfort**	⬭	⬭
**Sudden trouble seeing in one or both eyes**	⬭	⬭
**Pain or discomfort in the arms or shoulders**	⬭	⬭
**Shortness of breath**	⬭	⬭

24. Where did you learn about heart disease? *

Mark only one oval.

- School
- Tv
- Family and friends
- Doctors/nurses/other health professionals
- Newspapers and magazines
- Internet
- Other: ______________________

This content is neither created nor endorsed by Google.

Google Forms
