# Supplementary material for: Knowledge of cardiovascular disease risk factors among caregivers of cardiology patients attending Jordan University Hospital
Source: PeerJ. 2024 Jan 31;12:e16830. doi: 10.7717/peerj.16830 (PMC10838082; doi:10.7717/peerj.16830)
Supplement: Supplemental Information 3 [file peerj-12-16830-s003.pdf]

المعرفة بعوامل خطر الإصابة بأمراض القلب والأوعية الدموية بين القائمين على رعاية مرضى القلب في مستشفى الجامعة الأردنية

نحن مجموعة من الطلاب في الجامعة الأردنية -
كلية الطب نقوم بإجراء هذه الدراسة المقطعية ، والتي تهدف إلى تقييم معرفة وفهم
عوامل الخطر لأمراض القلب التاجية وأعراضها بين
القائمين على رعاية المرضى الخارجيين الذين يراجعون عيادات القلب في مستشفى الجامعة الأردنية في الأردن.

إكمال هذا
الاستبيان يحتاج إلى 5 دقائق فقط

جميع المعلومات
المقدمة ستبقى سرية وسوف تستخدم لأغراض البحث العلمي فقط.

* Indicates required question

1. هل توافق على المشاركة في هذه الدراسة *

Mark only one oval.

- نعم
- لا

Untitled Section

2. اسم الباحث

Mark only one oval.

- زيد
- اسامة
- اية
- باسل
- عطاري
- خطيب

3. العمر *

4. الجنس *

Mark only one oval.

- ذكر
- أنثى

5. المستوى التعليمي *

Mark only one oval.

- لا تعليم أساسي
- المرحلة الأساسية
- المرحلة الثانوية
- بكالوريوس أو أعلى

6. الحالة الزوجية *

Mark only one oval.

- أعزب
- متزوج
- مطلق
- أرمل

7. الوظيفة *

Mark only one oval.

- لا أعمل
- طالب
- موظف
- أعمال حرة
- متقاعد

8. الدخل الشهري *

Mark only one oval.

- أقل من 500
- 500-1000
- 1000-1500
- 1500-2000
- أكثر من 2000

9. مكان السكن *

Mark only one oval.

- مدينة
- قرية

10. علاقتك مع المريض الذي ترعاه/تصاحبه *

Mark only one oval.

- زوج
- ابن
- شقيق
- والد
- صديق

11. هل لديك تأمين صحي *

Mark only one oval.

- نعم
- لا

12. كيف تصف حالتك الصحية *

Mark only one oval.

- جيدة
- متوسطة
- سيئة

13. متى كانت آخر مرة قمت بفحوصات دورية للصحة العامة *

Mark only one oval.

- لم أقم بفحوصات أبدًا
- خلال سنة
- خلال أكثر من سنة

14. هل لديك أي من الأمراض الآتية *

Check all that apply.

- [ ] أمراض القلب والأوعية الدموية مثل ألسكتة القلبية
- [ ] سكتة دماغية
- [ ] ارتفاع ضغط الدم
- [ ] السكري
- [ ] أمراض الكلى المزمنة
- [ ] أمراض تنفسية مزمنة
- [ ] سرطان
- [ ] لا

15. هل هناك تاريخ عائلي لمرض القلب التاجي *

Mark only one oval.

- نعم
- لا
- لا أعلم

16. هل هناك تاريخ عائلي لوفيات إناث أقل من ٦٥ سنة أو ذكور أقل من ٥٥ سنة بسبب أمراض القلب *

Mark only one oval.

- نعم
- لا
- لا أعلم

17. هل أنت مدخن *

Mark only one oval.

- لا لم أدخن أبدًا
- نعم (على الأقل سيجارة واحدة في آخر 6 أشهر)
- مدخن سابق (لم أدخن في آخر 6 أشهر)

18. كم يوم تمارس الرياضة في الأسبوع *

Mark only one oval.

- لا أمارس الرياضة
- Other: ____________

19. كم دقيقة تمارس الرياضة في اليوم الواحد *

Mark only one oval.

- لا أمارس الرياضة
- Other: ____________

20. الوزن *

____

21. الطول *

22. كم تقدر قلقك من احتمالية الإصابة بسكتة قلبية *

Mark only one oval.

- لست قلقًا أبدًا
- قلق بنسبة ضئيلة
- شديد القلق

23. أجب عن الأسئلة التالية بناءً على معرفتك بأمراض القلب التاجية *

Mark only one oval per row.

	نعم	لا
**يمكن الشفاء من أمراض القلب التاجية عند إكمال جرعة الدواء الموصوفة**	◯	◯
**أمراض القلب هي السبب الرئيسي في الوفاة عالميًا**	◯	◯
**التدخين لا يزيد من نسبة حدوث أمراض القلب**	◯	◯
**داء السكري يزيد من نسبة حدوث أمراض القلب**	◯	◯
**ارتفاع ضغط الدم هو عامل مساعد في حدوث أمراض القلب**	◯	◯
**يعد الإكثار من شرب الكحوليات خطرًا على صحة الجهاز القلبي والوعائي**	◯	◯

ارتفاع الكوليسترول في الدم يحمي من الإصابة بأمراض القلب		
استهلاك الكثير من الخضروات والفواكه يزيد من احتمالية الإصابة بأمراض القلب		
استهلاك الكثير من ملح الطعام يشكل خطرًا على الجهاز القلبي والوعائي		
الوزن الزائد يزيد من احتمالية الإصابة بأمراض القلب		
ممارسة الرياضة بانتظام تقلل من احتمالية إصابة الشخص بأمراض القلب		
يعد كبر السن عامل خطر للإصابة بأمراض القلب		

بأمراض القلب

الرجال عرضة أكثر للإصابة بأمراض القلب مقارنة بالنساء

لا يمكن الوقاية من أمراض القلب والأوعية الدموية

التوقف عن التدخين يقلل احتمالية الإصابة بسكتة قلبية

البروتين الدهني عالي الكثافة (HDL) يشير إلى الكوليسترول الجيد و البروتين الدهني قليل الكثافة (LDL) يشير إلى الكوليسترول السيء

يعتبر ضغط 140/90 ارتفاعًا في ضغط الدم

يمكن لغير المدخنين أن يموتوا من التدخين السلبي

24. هل تعتقد أن الشكاوى التالية هي أعراض لنوبة قلبية؟ *

Mark only one oval per row.

	نعم	لا
ألم أو انزعاج في الصدر	⬭	⬭
ألم أو إزعاج في الفك أو الرقبة أو الظهر	⬭	⬭
ألم أو إزعاج في الذراعين أو الكتفين	⬭	⬭
ضيق في التنفس	⬭	⬭
الشعور بالضعف أو الدوار أو الإغماء	⬭	⬭
صعوبة مفاجئة في الرؤية بإحدى العينين أو كلتيهما	⬭	⬭

25. ما هو مصدر معلوماتك عن أمراض القلب *

Check all that apply.

- [ ] الأطباء / الممرضين / غيرهم من المهنيين الصحيين
- [ ] الأصدقاء أو العائلة والأقارب
- [ ] الانترنت
- [ ] التلفاز
- [ ] الجرائد أو المجلات
- [ ] Other: ______________________

This content is neither created nor endorsed by Google.

Google Forms
